# Association between Lipid Ratios and Insulin Resistance in a Chinese Population

**DOI:** 10.1371/journal.pone.0116110

**Published:** 2015-01-30

**Authors:** Liying Zhang, Shanying Chen, Aiwen Deng, Xinyu Liu, Yan Liang, Xiaofei Shao, Mingxia Sun, Hequn Zou

**Affiliations:** 1 Department of Nephrology, Third Affiliated Hospital of Southern Medical University, Guangzhou, Guangdong, China; 2 Department of Nephrology, First Affiliated Hospital of Inner Mongolia Medical University, Huhehaote, China; 3 Department of Nephrology, Zhangzhou Affiliated Hospital of Fujian Medical University, Zhangzhou, Fujian, China; 4 Department of Rehabilitation, Third Affiliated Hospital of Southern Medical University, Guangzhou, Guangdong, China; NIH / NIDDK, UNITED STATES

## Abstract

**Aim:**

To explore the association of lipid ratios and triglyceride (TG) with insulin resistance (IR) in a Chinese population. We also provide the clinical utility of lipid ratios to identify men and women with IR.

**Methods:**

This cross-sectional study included 614 men and 1055 women without diabetes. Insulin resistance was defined by homeostatic model assessment of IR > 2.69. Lipid ratios included the TG/ high density lipoprotein cholesterol (HDL-C), the total cholesterol (TC)/HDL-C and the low density lipoprotein cholesterol (LDL-C)/HDL –C. Logistic regression models and accurate estimates of the area under the receiver operating characteristic (AUROC) curves were obtained.

**Results:**

In normal-weight men, none of lipid ratios nor TG was associated with IR. In overweight/obese men, normal-weight women and overweight/obese women, the TG/HDL-C, the TC/HDL-C and TG were significantly associated with IR, and the associations were independent of waist circumference. All of the AUROCs for the TG/HDL-C and TG were > 0.7. The AUROCs for TC/HDL-C ratio were 0.69–0.77. The optimal cut-offs for TG/HDL-C were 1.51 in men and 0.84 in women. The optimal cut-offs for TG were 1.78 mmol/L in men and 1.49 mmol/L in women, respectively. In men, the optimal cut-off for LDL-C/HDL-C is 3.80. In women, the optimal cut-off for LDL-C/HDL-C is 3.82.

**Conclusion:**

The TG/HDL-C, the TC/HDL-C and TG are associated with IR in overweight/obese men, normal-weight and overweight/obese women. The LDL-C/HDL-C is only associated with IR in normal-weight women. The TG/HDL-C and TG might be used as surrogate markers for assessing IR.

## Introduction

Insulin resistance (IR) is a major risk of type 2 diabetes and cardiovascular diseases [1]. The gold standard method to detect IR, the hyperinsulinemiceuglycemic clamp, is impractical in clinical practice [2]. Previous studies have reported the triglyceride/high density lipoprotein cholesterol (TG/HDL-C) ratio is a surrogate marker of IR [3–9]. This might be a simple and reliable method to assess IR. However, the association of the TG/HDL-C ratio with IR might be ethnicity-dependant [6, 8, 10]. The association of the TG/HDL-C ratio with IR is demonstrated in Caucasian individuals [3–8]. One previous study based on African Americans did not support using TG or the TG/HDL-C ratio as an appropriate surrogate marker of IR [6]. Another study also indicated that the TG/HDL-C ratio was not associated with IR in South Asian women [8]. Limited studies indicated that the TG/HDL-C ratio might be a surrogate marker of IR in Chinese population [9, 10, 11]. In these two studies, the sample sizes were relatively small and one study was limited to non-obese women [10, 11]. In another study based on 812 Chinese subjects, it was indicated that using the TG/HDL-C ratio can significantly enhance the diagnostic accuracy of IR defined by homeostatic model assessment of insulin resistance (HOMA-IR) [9]. Therefore, using the TG/HDL-C ratio as a surrogate marker of IR has not been widely described in Chinese population. Lipid ratios can also be used to assess the risk of cardiovascular diseases and chronic kidney disease [12,13,14].

Apart from the TG/HDL-C ratio, other lipid ratios or TG might also be used to predict IR. One study indicated that the low density lipoprotein cholesterol (LDL-C)/HDL-C ratio might be the best reliable marker of IR in non-obese Japanese adults [15]. TG has been reported to be an alternative surrogate for IR [6]. Our previous study also indicated that serum TG is a suitable predictor for CKD in men [14].

The aim of the present study is to explore the associations of lipid ratios and TG with IR in a Chinese population. We also provide the clinical utility of lipid ratios to identify men and women with IR.

## Methods

### Study population

All of data were drawn from a population-based, cross-sectional survey in Southern China. We have described the epidemiology study elsewhere [16]. The study was conducted between June, 2012, and Oct, 2012, on Wanzhai Town, Zhuhai City. 1843 subjects had complete data. In the present analysis, we excluded subjects with diabetes. Diabetes was defined as having a history of diabetes or fasting glucose ≥ 7.0mmol/Lor 2-hour postprandial blood glucose ≥ 11.1mmol/L [17]. 115 Subjects having history of diabetes and 36 subjects with fasting blood glucose ≥ 7.0mmol/L were excluded. All subjects with fasting glucose > 5.6 mmol/L received a 2-hour postprandial blood glucose test. Subjects were given 75 grams of glucose solution to drink within a 5 minute and 2 hours late blood samples were collected for measurement of blood glucose. We also excluded 14 subjects with 2-hour postprandial blood glucose ≥ 11.0mmol/. Finally, 1669 subjects with mean of age 52 ± 16 were included in the present analysis.

This study has been approved by the ethics committee of the Third Affiliated Hospital of Southern Medical University. Written informed consents were obtained. All of the work has been carried out in accordance with the Declaration of Helsinki.

### Assessment of IR

We used HOMA-IR to assess IR. Homeostatic model assessment of insulin resistance was calculated as the following formula: fasting plasma glucose (mmlo/L) × fasting insulin (IU/L)/22.5 [18]. Using blood samples collected after a minimum 10 hour fasting, fasting glucose was measured by calorimetric methods, and fasting insulin was measured using electrochemiluminescence immunoassays [16]. Based on an epidemiology survey in China, IR was defined as HOMA-IR > 2.69 (exceeding the 75% percentile of HOMA-IR in normal glucose tolerance subjects) [19].

### Serum Lipid Profiles

Fasting serum lipids were determined using blood specimens after an overnight fasting for at least 10 hours. Using calorimetric methods with the Roche assay (Roche cobas6000), serum total cholesterol (TC), serum TG, and serum HDL- C were determined [14]. Low density lipoprotein cholesterol was indirectly calculated using the Friedewald Equation [20]. We calculated three lipid ratios: the TG/HDL ratio, the TC/HDL ratio and the LDL/HDL ratio.

### Confounders

Information on age, sex, education attainment, personal and family health history and lifestyle habits was obtained by questionnaires [16]. According to years of schooling, we divided education status into two categories: (1) high senior school or above (2) 0 years of schooling to junior middle school. Physical inactivity was defined as less than one hour of moderately intense activity per week. We used a calibrated mercury sphygmomanometer to determine blood pressures in the right arm of subjects after a rest period of 5 minutes. Blood pressure was measured three times and the average of the three consecutively measurement was calculated [16].

Anthropometric measurements including height, weight, and waist circumference were measured by the World Health Organization recommended protocols [21]. Waist circumference was accurate to 0.1cm and weight was accurate to 0.1 kg. Body mass index (BMI) was calculated as weight (in kilograms) divided by the square of the height (in meters).

### Data analysis

All statistical analyses were performed using Stata (version 11). One previous study indicated that the association of the TG/HDL-C ratio with IR might be sex-dependant [8]. Body fat accumulates differently in men and women, and we also use different waist circumference cut-offs for men and women in clinical practice [22]. The cut-offs for the TG/HDL-C ratio also differ in men and women [23]. In the present study, all statistical analyses were run in men and women separately. Descriptive statistics for continuous variables were presented as mean ± standard deviation if variables were normally distributed or as median and interquartile range if variables were skewed distributed. Frequencies and percentages were used to express categorical variables. A p-value of <0.05 was considered significance.

Insulin resistance exists not only in obese individuals but also in individuals with normal weight [24]. The mechanics of IR in normal-weight individuals have not been clarified, and fat mass might be associated with IR in normal-weight subjects [25]. The results of one previous study indicated that the TG/HDL-C ratio might be a surrogate for IR in non-obese Chinese women [11]. In the present study, we divided both men and women into two different BMI categories: a normal-weight subgroup and an overweight/obese subgroup. Based on a previous meta-analysis, the optimal cut-off for BMI is 24 kg/m^2^ in Chinese population [22]. Clinical characteristics of subjects in different BMI categories were presented and compared using Student t test or rank-sum test for continuous variables and the chi-squared test or Fisher’s exact test for categorical variables.

To explore whether lipid ratios and TG are associated with IR in Chinese individuals, logistic regression models were used and odds ratios (ORs) and 95% confidence interval (CI) were calculated. Lipid ratios and TG were used as an independent variable, respectively. Insulin resistance was defined as HOMA-IR > 2.69 and insulin sensitivity was defined if HOMA-IR < or = 2.69 [19]. In the adjusted models, potential confounders were added. These confounders included socio-demographic status (age and educational attainment), lifestyle factors (current smoking, current alcohol use, and physical inactivity), waist circumference, systolic blood pressure and diastolic blood pressure. The prior study indicated waist circumference is associated with IR even in normal-weight individuals [25], so waist circumference was also added as a covariate.

We used HOMA-IR as a continuous variable to examine the correlations of HOMA-IR with the lipid ratios and TG. The models were adjusted for age, educational attainment, current smoking, current alcohol use, physical inactivity, waist circumference, systolic blood pressure and diastolic blood pressure. All variables with a skewed distribution including HOMA-IR, TG, the TG/HDL-C ratio, the TC/HDL-C ratio and the LDL-C/HDL-C ratio were logarithmically transformed in the regression models.

We also run the full models to explore the associations of TG/HDL-C ratio with IR in the whole population. Next, sex*TG/HDL-C ratio and BMI category* TG/HDL-C ratio were added into the adjusted models, respectively. The p-values for the interaction terms of sex* TG/HDL-C ratio and BMI category* TG/HDL-C ratio in the full models of IR were explored.

Accurate estimates of the area under the receiver operating characteristic (AUROC) curve analysis was conducted by using the TG/HDL-C ratio, the TC/HDL-C ratio, the LDL-C/HDL-C ratio, and TG as continuous variables in the logistic regression models. Accurate estimates of AUROC were obtained. Insulin resistance is mostly caused by abdominal obesity and waist circumference is used as an alternative method to assess IR [6, 26]. So in the present study, the AUROC of waist circumference is assigned as a standard AUROC. The AUROCs were also adjusted for covariates used in the logistic models. Based on the AUROCs, the diagnostic value of lipid ratios and TG were assessed: an AUROC of 0.5 was considered as no discrimination, an AUROC between 0.7 and 0.8 was considered as acceptable, an AUROC between 0.7 and 0.8 was considered as excellent, and an AUROC ≥0.9 was considered as outstanding [27]. We also used another anthropometric index, BMI, as a continuous variable in the logistic regression models to examine whether BMI is a better predictor of IR than waist circumference and the lipid ratios.

Youden’s index was calculated as (specificity + sensitivity −1) and used to select the optimal cut-offs for each lipid ratio and TG.

## Results

### Clinical characteristics of three subgroups in different CRP categories (Table 1)

Characteristics of male and female subjects in different BMI categories were presented in Table 1. In both men and women, overweight/obese subjects had a larger waist circumference and higher levels of lipid ratios than normal-weight subjects. Compared with the normal-weight subjects, overweight/obese subjects had significantly higher levels of lipid ratios and TG. Overweight/obese subjects also had higher blood pressures and higher levels of fasting glucose, and the differences were significant.

**Table 1:** Characteristics ^a^ of men and women by different BMI categories.

	**Men**			**Women**		
	**Normal-weight**	**Overweight/obesity**	**P value**	**Normal-weight**	**Overweight/obesity**	**P value**
	**n = 343**	**n = 271**		**n = 685**	**n = 370**	
Age (years)	52.6 ± 16.1	52.2 ± 13.2	0.75	49.8 ± 15.1	54.9 ± 12.0	< 0.001
Body Mass Index (kg/m2)	21.4 ± 2.0	25.8 ± 2.3	<0.001	20.9 ± 2.0	26.5 ± 2.3	<0.001
Waist circumference(cm)	80.3 ± 7.4	93.2 ± 6.7	<0.001	76.0 ± 7.3	88.5 ± 7.9	<0.001
Current smoker (%)	112 (32.65)	87 (32.10)	0.91	9 (1.31)	0	0.03
Current alcohol use (%)	46 (13.41)	37 (13.65)	0.93	7 (1.02)	4 (1.08)	1
Education attainment High school or above (%)	182 (53.06)	131 (48.34)	0.25	281 (41.02)	113 (30.54)	0.001
Physical inactivity (%)	176 (51.31)	166 (61.25)	0.02	403 (58.83)	201 (54.32)	0.16
Systolic blood pressure (mm Hg)	127.1 ± 19.9	133.4 ± 17.2	< 0.001	122.3 ± 19.1	133.4 ± 20.7	<0.001
Diastolic blood pressure (mmHg)	77.3 ± 10.9	82.2 ± 9.9	< 0.001	74.5 ± 10.6	80.9 ± 10.7	<0.001
Fasting glucose (mmo/l)	4.70 ± 0.50	4. 91 ± 0.64	< 0.001	4.65 ± 0.44	4.90 ± 0.56	<0.001
Fasting insulin (uU/mL)	6.38 (4.48–8.88)	11.43 (7.67–16.58)	<0.001	7.14 (5.17–9.64)	10.64 (7.98–15.27)	<0.001
HOMA – IR (uU/ml .mmol/mL)	1.37 (0.91–1.87)	2.50 (1.64–3.65)	<0.001	1.47 (1.08–2.09)	2.30 (1.70–3.33)	<0.001
Serum Triglyceride (mmol/L)	1.14 (0.83–1.63)	1.78 (1.17–2.41)	<0.001	1.00 (0.75–1.39)	1.38 (0.99–1.94)	<0.001
LDL (mmol/L)	3.10 ± 0.92	3.20 ± 0.90	0.22	3.08± 0.87	3.38 ± 0.90	<0.001
HDL (mmol/L)	1.51 ± 0.33	1.35 ± 0.26	<0.001	1.64 ± 0.33	1.51 ± 0.27	<0.001
TG/HDL-C ratio	0.79 (0.53–1.21)	1.35(0.87–1.95)	<0.001	0.61 (0.42–0.96)	0.91(0.63–1.38)	<0.001
TC/HDL-C ratio	3.56 (2.97–4.10)	4.14(3.63–4.55)	<0.001	3.21 (2.74–3.75)	3.78(3.27–4.76)	<0.001
LDL/HDL-C ratio	2.09 (1.66–2.52)	2.37(1.99–2.86)	<0.001	1.88 (1.49–2.30)	2.22(1.85–2.64)	<0.001

^a^ Mean ± SD or median (25th to 75th percentiles) for continuous variables and absolute and relative (%) values for category variables are presented.

HOMA-IR: Homeostatic model assessment of insulin resistance; TC: total cholesterol; TG: triglyceride; HDL-C: high density lipoprotein cholesterol; LDL-C: low density lipoprotein cholesterol

Analyses were used to explore the differences in study characteristics between normal and overweight subjects within each sex

### Associations of lipid ratios and TG with IR defined by HOMA-IR in different BMI categories (Table 2)

The association of the TG/HDL-C ratio and IR was first explored in the whole population. Next, sex*TG/HDL-C ratio and BMI category*TG/HDL-C ratio were added into the adjusted models, respectively. The interaction term of sex*TG/HDL-C ratio is 0.1 in the full model of insulin resistance and that of BMI category*TG/HDL-C ratio is <0.001.

**Table 2:** Associations ^a^ of lipid profiles and triglycerides with insulin resistance in men and women categorized by BMI phenotype.

	**TG/HDL-C ratio**		**TC/HDL-C ratio**		**LDL-C/HDL-C ratio**		**Triglyceride**	
	**OR (95% CI)**		**OR (95% CI)**		**OR (95% CI)**		**OR (95% CI)**	
Normal-weight Men *	1.64 (0.97–2.77)	0.06	0.96 (0.55–1. 70)	0.90	0.96 (0.51–1.82)	0.91	0.96 (0. 51–1.82)	0.91
Overweight /obese Men *	1.80 (1.28–2.53)	0.001	1.67 (1.11–2.53)	0.01	1.03 (0.68–1.57)	0.88	1.36 (1.06–1.75)	0.02
Normal-weight Women *	3.17 (2.07–4.86)	< 0.001	1.67 (1.20–2.32)	< 0.001	1.55 (1.01–2.38)	0.046	2.62 (1.84–3.73)	< 0.001
Overweight /obese Women *	2.85 (1.89–4.31)	< 0.001	2.01 (1.41–2.97)	< 0.001	1.38 (0.94–2.01)	0.099	2.02 (1.47–2.76)	< 0.001

**^a^** TC: total cholesterol; TG: triglyceride; HDL-C: high density lipoprotein cholesterol; LDL-C: low density lipoprotein cholesterol

* Adjusted for age, current smoker, current alcohol use, physical inactivity, education attainment, blood pressure and waist circumference

The TG/HDL-C ratio, the TC/HDL-C ratio and TG were associated with IR in overweight/obese men and both normal-weight and overweight/obese women. The LDL-C/HDL-C ratio was associated with IR in normal—weight women. In normal-weight men, none of lipid ratios nor TG was associated with IR.

In normal-weight men, none of lipid ratios nor serum TG was associated with IR in the adjusted models. In overweight/obese men, the TG/HDL-C ratio, the TC/HDL-C ratio and TG were significant associated with IR, and the associations were independent of waist circumference and other potential confounders. The OR was 1.80 (95% CI 1.28–2.53, P = 0.001) for one unit increase in the TG/HDL-C ratio, 1.67 (95% CI 1.11–2.53, P = 0.01) for one unit increase in the TG/HDL-C ratio, or 1.36 (95% CI 1.06–1.75, P = 0.02) for one mmol/L increase in TG, respectively.

In normal-weight women, all of lipid ratios and TG were significantly associated with IR and the associations were independent of other confounders. Every unit increase in the TG/HDL-C ratio was associated with OR 3.17 (95% CI 2.07–4.86, P < 0.001). Every unit increase in the TC/HDL-C ratio was associated with OR 1.67 (95% CI 1.20, 2.32, P = 0.001). Every unit increase in the LDL-C/HDL-C ratio was associated with OR 1.55 (95% CI 1.01, 2.38, P = 0.046). One mmol/L increase in TG was associated with OR 2.62 (95% CI 1.84, 3.73, P < 0.001).

In overweight/obese women, the TG/HDL-C ratio, the TC/HDL-C ratio, and TG were associated with IR. The OR for the TG/HDL-C ratio (every unit increase) was 2.85 (95% CI 1.89–4.31, P < 0.001). The OR for the TC/HDL-C ratio (every unit increase) was 2.01 (95% CI 1.41–2.97, < 0.001). The OR for TG (each mmol/l increase) was 2.02 (95% CI 1.47–2.76, P < 0.001), respectively.

When HOMA-IR was used as a continuous variable in the adjusted regression models, TG, the TG/HDL-C ratio and the TC/HDL-C ratio were significantly association with HOMA-IR in both the normal-weight or overweight/obese men and women (P < 0.05). The Pearson correlation coefficient between TG and HOMA-IR was 0.32–0.37. The Pearson correlation coefficient between the TG/HDL-C ratio and HOMA-IR was 0.33–0.41. The Pearson correlation coefficient between the TC/HDL-C ratio and HOMA-IR was 0.24–0.33. The LDL-C/HDL-C ratio was only associated with HOMA-IR in normal-weight women (The Pearson correlation coefficient was 0.07–0.22).

### Comparison of AUROCs for potential markers of IR in different BMI categories by sex (Table 3)

**Figure 1:**
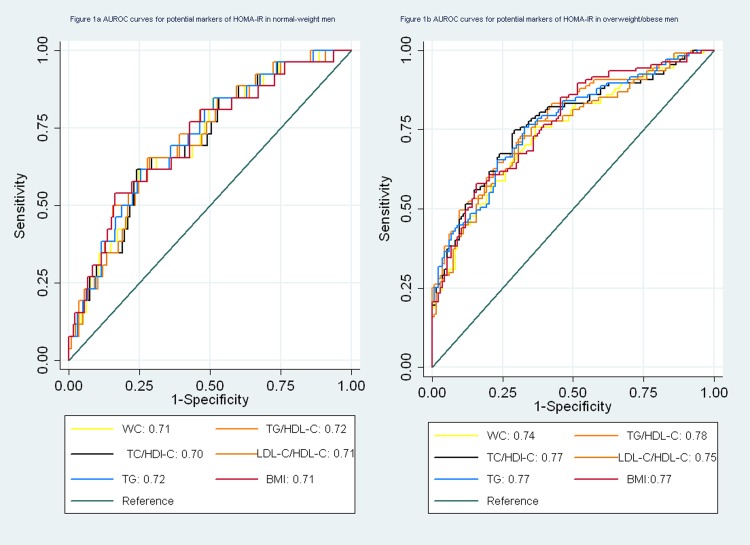
a. AUROC curves for potential markers of HOMA-IR in normal-weight men. b. AUROC curves for potential markers of HOMA-IR in overweight/obese men.

In logistic regression models, waist circumference was associated with IR in normal-weight men (not shown in the table). The AUROC for waist circumference was 0.71 (95% CI 0.61–0.81) (Fig. 1A). In overweight/obese men, the AUROC curve analyses showed that the TG/HDL ratio, the TC/HDL-C ratio and serum TG were acceptable predictors for IR defined by HOMA-IR (the AUROC > 0.7) (Fig. 1B).

**Table 3:** Area under the receiver operating characteristic curves ^a^ for potential markers of HOMA-IR.

	**WC**	**BMI**		**TG/HDL-C**		**TC/HDL-C**		**LDL-C/HDL-C**		**Triglyceride**	
	**AUC (95% CI)**	**AUC (95% CI)**	**P value**	**AUC (95% CI)**	**P value**	**AUC (95% CI)**	**P value**	**AUC (95% CI)**	**P value**	**AUC (95% CI)**	**P value**
**Normal-weight men (Fig-1A)** *	0.71 (0.61–0.81)	0.71 (0.61–.82)	0.62	0.72 (0.62–0.82)	0.26	0.70 (0.60–0.81)	0.64	0.71 (0.61–0. 81)	0.96	0.72 (0.62–0.82)	0.26
**Overweight /obese men (Fig-1B)** *	0.74 (0.68–0.81)	0.77 (0.71–0.83)	0.17	0.78 (0.72–0.84)	0.05	0.77 (0.71–0.73)	0.11	0.75 (0.69–0.81)	0.52	0.77 (0.71–0.83)	0.11
**Normal-weight women (Fig-2A)** *	0.72 (0.66–0.78)	0.73 (0.66–0.79)	0.49	0.77 (0.71–0.83)	0.002	0.74 (0.68–.80)	0.09	0.73 (0.66–0.79)	0.30	0.77 (0.71–0.83)	0.003
**Overweight/obese women (Fig 2B)** *	0.64 (0.57–0.70)	0.64 (0.58–0.71)	0.65	0.73 (0.68–0.79)	< 0.001	0.69 (0.64–0.75)	0.04	0.65 (0.59–0.71)	0.53	0.71 (0.66–0.77)	0.005

* Adjusted for age, current smoker, current alcohol use, physical inactivity, education attainment, blood pressure, and waist circumference

**^a^** WC: waist circumference; BMI body mass index; TC: total cholesterol; TG: triglyceride; HDL-C: high density lipoprotein cholesterol; LDL-C: low density lipoprotein cholesterol; HOMA-IR: Homeostatic model assessment of insulin resistance

**Figure 2:**
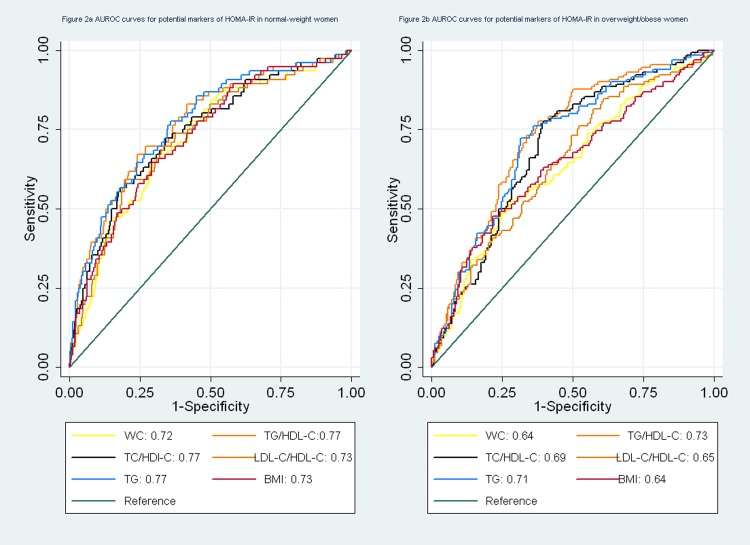
a. AUROC curves for potential markers of HOMA-IR in normal-weight women. b. AUROC curves for potential markers of HOMA-IR in overweight/obese women.

In normal-weight women, waist circumference, the TG/HDL-C ratio, the TC/HDL-C ratio, the LDL-C/HDL-C ratio and TG were suitable predictors for IR. Among four variables, the TG/HDL-C ratio and TG were better predictors for IR than waist circumference and the TC/HDL-C ratio. The TG/HDL-C ratio and TG had significantly higher AUROCs than waist circumference (P < 0.05) (Fig. 2A). In overweight/obese women, TG and the TG/HDL-C ratio were acceptable predictors for IR (the AUROC >0.70). The AUROCs for waist circumference and the LDL-C/HDL-C ratio were significantly lower (0.6 < AUROC <0.7) (Fig. 2B).

BMI was also a suitable predictor for IR in men and normal-weight women (the AUROC > 0.7), but it was not better than waist circumference, TG and the TG/HDL-C ratio (Table 3).

### Optimal cut-offs for lipid ratios and TG in male and female subjects (Table 4)

The optimal cut-offs for lipid ratios and TG are listed in Table 4. The cut-offs for TG/HDL-C ratio were 1.51 in men and 0.84 in women. The cut-offs for TG were 1.78 in men and 1.49 in women, respectively. In men, the optimal cut-off for LDL-C/HDL-C ratio was 3.80. In women, the optimal cut-off for LDL-C/HDL-C ratio was 3.82.

**Table 4:** Optimal cut-offs for lipid ratios and the associated sensitivities and specificities^a^.

	**Cut-point**	**Sensitivity (%)**	**Specificity (%)**
**Men**			
**Waist circumference (cm)**	87	80.8	62.4
**BMI (kg/m2)**	25.15	68.49	80.09
**TG/HDL-C ratio**	1.51	54.1	78.2
**TC/HDL-C ratio**	3.80	76.0	57.1
**LDL-C/HDL-C ratio**	1.90	54.1	75.9
**Triglyceride (mmol/L)**	1.78	57.5	72.4
**Women**			
**Waist circumference (cm)**	81	77.16	60.02
**BMI (kg/m2)**	23.44	72.29	67.11
**TG/HDL-C ratio**	0.84	72.4	69.0
**TC/HDL-C ratio**	3.82	57.8	76.1
**LDL-C/HDL-C ratio**	2.26	71.8	68.0
**Triglyceride (mmol/L)**	1.49	55.6	79.7

**^a^** Adjusted for age, current smoker, current alcohol use, physical inactivity, education attainment, blood pressure and waist circumference

The cut-offs for waist circumference were 87 cm in men and 81 cm in women. Men and women had different cut-offs for BMI (25.15 kg/m^2^ in men and 23.44 kg/m^2^ in women).

## Discussion

The results of the present study indicated that the TG/HDL-C ratio, the TC/HDL-C ratio and TG were associated with IR in overweight/obese men, normal-weight women and overweight/obese women. Both of the AUROCs for the TG/HDL-C, and TG were > 0.7. The AUROCs for TC/HDL-C ratio were 0.69–0.77. The LDL-C/HDL-C ratio might not be associated with IR in both men and overweight women. In normal-weight men, none of lipid ratios nor TG was associated with IR.

Both elevated TG and low HDL-C are components of metabolic syndrome [23]. In 1997, it was reported by Gaziano et al [28] that the TG/HDL-C ratio was a strong predictor of myocardial infarction. In 2003, McLaughlin et al reported that serum TG and the TG/HDL-C ratio were the most useful metabolic markers in predicting IR [3]. In this study, TG or the TG/HDL-C ratio was recommended as the most practical approach to identify IR in overweight individuals [3]. Using the level of the TG/HDL-C ratio (≥ 3.5) might be a simple method to identify insulin-resistant, dyslipidemia patients [4]. The results of one study based on 770 non-Hispanic whites, 243 non-Hispanic blacks, and 346 Mexican Americans also supported using the TG/HDL-C ratio as a simple and useful predictor for hyperinsulinemia among non-diabetic individuals. The TG/HDL-C ratio was significantly associated with hyperinsulinemia regardless of race/ethnicity [5].

Subsequent studies indicated that the association of the TG/HDL- C ratio with IR might differ by ethnicity and the TG/HDL-C ratio should not be widely used as a suitable predictor of IR in all ethnic groups [6, 8]. In Kim-Dorner et al’s study [6], 50 Caucasian and 99 African Americans were included. The results indicated that both TG and the TG/HDL-C were not appropriate predictors for IR defined by HOMA-IR in African Americans ( an AUC < 0.70) [6]. The TG/HDL-C ratio is also not a good surrogate marker of IR for Hispanics obese youth and South Asian women [7, 8]. As we know, there are limited evidences which support using the TG/HDL-C as a surrogate marker of IR in Chinese individuals. In the present analysis, the results indicated that both the TG/HDL-C ratio and TG can be used as an appropriate predictor for IR in women and overweight/obese men but not in normal-weight men. In the logistic models, when waist circumference and the TG/HDL-C ratio were added into the same models, the AUROCs were significantly increased (P < 0.001). The result indicated that using the TG/HDL-C ratio might increase the diagnostic accuracy of IR. The finding was consistent with a previous study based on 812 Taiwanese adults [9].

Besides LDL-C and HDL-C which are the primary targets of therapy in most clinical guidelines, other lipoprotein-lipid such as non-HDL-C or apolipoprotein B (apoB) could provide a predictive value [13]. The TC /HDL-C ratio and the LDL-C/HDL-C ratio are also used for assessing cardiovascular risks [13,14]. Although studies on the association of the TC/HDL-C ratio or the HDL-C /HDL-C ratio with IR were relatively less, the results support using the TC/HDL-C ratio or the HDL-C /HDL-C ratio as a surrogate marker of IR [15, 29, 30]. A study based on non-diabetic individuals with impaired fasting glucose showed that the AUROC of the TC/HDL-C ratio for predicting IR was 0.78 (95% CI, 0.65–0.91) [29]. The TC/HDL-C ratio also might have higher sensitivity and specificity in diagnosing polycystic ovarian syndrome with IR [30]. Previous studies based on small samples indicated that the LDL-C/HDL-C ratio might be a reliable marker of IR [15]. In the Quebec Cardiovascular Study [31], 2103 middle-aged men were included and the metabolic profiles were measured. It was concluded that variation in the TC/HDL-C ratio may be related to the IR syndrome than variation in the LDL-C/HDL-C ratio [31]. In a study based on 105 women with polycystic ovarian syndrome, the associations of the TC/HDL-C ratio or the LDL-C /HDL-C ratio with IR have been examined [30]. As we know, the associations have not been explored in Chinese men and women without polycystic ovarian syndrome. In the present analysis, the results indicated that the TC/HDL-C ratio also is associated with IR in overweight/obese men and both normal-weight and overweight/obese women. But the findings did not support using the LDL-C /HDL-C ratio as a predictor of IR in both men and women regardless of BMI categories.

In the present study, it is shown that the optimal cut-off for the TG/HDL-C ratio is 1.51 (3.48, when using mmol/L/mmol/L) for men and this result is similar to the previous study [4]. However, the optimal cut-off for TG/HDL-C ratio in women is 0.84. In one prior study based on Chinese individuals, it was shown that the best cut-off for the TG/HDL-C is 1.1. [10]. Based on one previous study, the optimal cut-offs for waist circumference are 85 cm in men and 80 cm in women in Chinese population [22]. In the present study, we also had similar optimal cut-offs for waist circumference (87 cm in men and 81 cm in women). The recommended optimal cut-off for BMI is 24 kg/m^2^ in men and women in Chinese population [22]. However, in the present study, we get difference optimal cut-offs for BMI in men and women (25.15 kg/m^2^ in men and 23.44 kg/m^2^ in women).

In the present study, we did not use the gold standard method, the hyperinsulinemiceuglycemic clamp, to detect IR. This is a major limitation of the present study. Several alternative methods can be used to detect IR. These methods included HOMA-IR, fasting insulin, Quantitative insulin sensitivity check index (QUICKI) [32], glucose/ insulin ratio [33] and so on. A previous study based on 15, 568 Chinese individuals provided information on the optimal cut-off for HOMA-IR in Chinese individuals [19]. In this national survey, subjects were recruited from 19 provinces of China [19]. Not enough evidence is available to determine optimal cut-offs for QUICKI and fasting serum insulin in the Chinese populations. A study based on 508 Chinese individuals with diabetes mellitus also indicated that that the fasting glucose/ insulin ratio is not a reliable index of insulin sensitivity [33].

In the present study, we found that in men with normal weight, none of the lipid ratios nor TG was a reliable marker of IR. Because the sample size is relatively small, the associations of lipid ratios and IG with IR need to be further explored. Waist circumference can be used to predict IR in normal-weight men, and the AUROC for waist circumference was 0.86. In our previous study, it was found that waist circumference is associated with IR and metabolic syndrome in normal-weight subjects [25]. But in that study, serum lipids were not added as covariates. In the present study, it was found that the association of waist circumference with IR in normal-weight men was independent of the TG/HDL ratio.

Other limitations of the present study include two additional points. This was only a cross-sectional study not a longitudinal study. The sample might be bias because only 37% of subjects were men. However, we have analyzed the data in men and women, respectively. The main advantages included using three lipid ratios including the TG/HDL-C ratio, the TC/HDL-C ratio and the LDL-C/HDL-C ratio and the sample was relatively large compared with previous studies [6, 9, 10, 11].

## Conclusion

The TG/HDL-C ratio, the TC/HDL-C ratio and TG are associated with IR in overweight/obese men and both normal-weight and overweight/obese women. All of the AUROCs for the TG/HDL-C, and TG were > 0.7. The AUROCs for TC/HDL-C ratio were 0.69–0.77. The LDL-C/HDL-C ratio should not be used as a reliable predictor for IR in both men and overweight women. In normal-weight men, none of the lipid ratios nor TG was associated with IR, and waist circumference is significantly associated with IR in normal-weight men. The TG/HDL-C ratio and TG might be recommended as surrogate markers of IR in overweight/obese men, normal-weight and overweight/obese women.
